# Mobility enhancement among older adults 75 + in rural areas: Study protocol of the MOBILE randomized controlled trial

**DOI:** 10.1186/s12877-021-02739-0

**Published:** 2022-01-20

**Authors:** Christine Haeger, Sandra A Mümken, Julie L O‘Sullivan, Robert P Spang, Jan-Niklas Voigt-Antons, Martin Stockburger, Dagmar Dräger, Paul Gellert

**Affiliations:** 1 Charité Universitätsmedizin Berlin corporate member of Freie Universität Berlin Humboldt Universität zu Berlin, Institute of Medical Sociology Rehabilitation Science, Charitéplatz 1, 10117 Berlin, Germany; 2 Technische Universität Berlin, Quality Usability Lab, Straße des 17. Juni 135, 10623 Berlin, Germany; 3 German Research Centre for Artificial Intelligence (DFKI), Alt-Moabit 91c, 10559 Berlin, Germany; 4 Havelland Kliniken Unternehmensgruppe, Ketziner Straße 19, 14641 Nauen, Germany

**Keywords:** Rural areas, Mobility in old age, Preventive home visit, Global positioning system (GPS)

## Abstract

**Background:**

Maintaining mobility in old age is crucial for healthy ageing including delaying the onset and progress of frailty. However, the extent of an individuals´ mobility relies largely on their personal, social, and environmental resources as outlined in the Life-Space Constriction Model. Recent studies mainly focus on facilitating habitual out-of-home mobility by fostering one type of resources only. The MOBILE trial aims at testing whether tablet-assisted motivational counselling enhances the mobility of community-dwelling older adults by addressing personal, social, and environmental resources.

**Methods:**

In the MOBILE randomized controlled trial, we plan to enrol 254 community-dwelling older adults aged 75 and older from Havelland, a rural area in Germany. The intervention group will receive a tablet-assisted motivational counselling at the participant´s home and two follow-up telephone sessions. Main focus of the counselling sessions lays on setting and adapting individual mobility goals and applying action planning and habit formation strategies by incorporating the personal social network and regional opportunities for engaging in mobility related activities. The control group will receive postal general health information. The primary mobility outcome is time out-of-home assessed by GPS (GPS.Rec2.0-App) at three points in time (baseline, after one month, and after three months for seven consecutive days each). Secondary outcomes are the size of the GPS-derived life-space convex hull, self-reported life-space mobility (LSA-D), physical activity (IPAQ), depressive symptoms (GDS), frailty phenotype, and health status (SF-12).

**Discussion:**

The MOBILE trial will test the effect of a motivational counselling intervention on out-of-home mobility in community-dwelling older adults. Novel aspects of the MOBILE trial include the preventive multi-level intervention approach in combination with easy-to-use technology. The ecological approach ensures low-threshold implementation, which increases the benefit for the people in the region.

**Trial registration:**

The MOBILE trial is prospectively registered at DRKS (Deutsches Register Klinischer Studien, German Registry of Clinical Trials) DRKS00025230. Registered 5 May 2021.

## Background

Due to current and future changes in demographic society distributions, more attention must be paid to the aspect of healthy ageing [1]. Healthy ageing is challenged as people get older is the growing prevalence of the frailty syndrome that varies from 4 to 59% depending on the used conceptualisation, assessments, and defined age group [2]. Frailty is characterised by reduced physical reserves, activity restrictions, and a low-level of social participation leading to a decreased ability to cope with everyday stressors, a higher risk of adverse health outcomes, and overall reduced mobility [3, 4]. Therefore, interventions to prevent or delay the onset of frailty that are easy to apply and scalable are crucially needed. One way of preventing or delaying frailty is the facilitation of out-of-home mobility [5], as it is a prerequisite for participation in physical activities, social in-person involvement, and access to health care [6, 7]. Out-of-home mobility can be can be defined as movement through outdoor living environments called life-spaces expanding from one´s home to the neighbourhood, city lived in, and beyond [7, 8].

According to the Life-Space Constriction model, a person´s out-of-home mobility is determined by a combination of personal (e.g., health status, health literacy, coping strategies), social (e.g., social network, connection to neighbourhood), and environmental (e.g., regional infrastructure, access to health care) resources and obstacles [9], indicating that multi-level interventions on facilitating out-of-home-mobility seem to be most promising [5, 9]. Although the Life-Space Constriction model proposes the combination of individual, social and environmental resources and although there is evidence from longitudinal studies on the relationship between social factors and mobility [10] most interventional studies largely relied on addressing personal resources only. Personal goal setting and motivational support seem to be promising components as highlighted in the AGNES trial [11]. This applies especially when used techniques are based on well-founded psychological theories such as coping with loss or onset of disability as well as self-regulatory strategies to change behaviour [12, 13]. There is little evidence about the use of behaviour change techniques to maintain or modify out-of-home mobility in interventions to prevent frailty and one promising but solemnly used technique may be the formation of routines/habits [14, 15]. In addition results of a recent intervention study in Finland suggest that more evidence is needed on the short- and long-term effects of face-to-face counselling interventions addressing participation in mobility-related activities [16]. However, in the Finnish study, out-of-home mobility was solely measured using self-reported instruments. There is a growing body of evidence that out-of-home mobility is measured more precisely using Global Positioning Systems (GPS) data rather than the commonly used self-reported questionnaires [17]. Additionally there is existing evidence that out-of-home mobility is notably restricted in those older adults living in rural areas due to less infrastructure, car-dependency, and difficult access to health care, indicating the need of intervention studies in rural areas [18]. Hence, little research has been done when it comes to combining individual goal setting with social and environmental resources in a multi-component intervention with objective GPS measurements, especially when focusing on prevention or reversing frailty in older adults in rural areas [5, 19, 20]. To close the defined research gaps, the MOBILE (Mobility in Old age By Integrating health care and personal network resources in older adults Living in rural arEas) trial is aiming to determine whether a motivational counselling intervention can facilitate out-of-home mobility in older adults living in rural areas.

### Objectives

The main objective of the MOBILE trial is to test the effectiveness of a motivational counselling intervention to improve out-of-home mobility and thereby possibly preventing or delaying frailty in community-dwelling older adults in a particular rural area. Furthermore, drawing on the Life-Space Constriction model, we will be investigating the resources at personal, social, and environmental level and their relationship with out-of-home mobility, which, in turn, will be related with indicators of health, wellbeing, and frailty.

## Methods/design

### Trial design

The MOBILE trial is a randomized controlled interventional study using a pragmatic sample comparing an intervention to facilitate out-of-home mobility with generic health and mobility information that apply to the participants living area. Data will be collected as Computer Assisted Personal Interview (CAPI) and Computer Assisted Telephone Interviews (CATI) on five points in time (T_0_ – T_4_) and will consist of questionnaires, assessments, and objective GPS measurements. At baseline (T_0_) a comprehensive questionnaire consisting of established valid scales focusing on personal, social, and environmental resources is conducted as CAPI by a trained study nurse. Furthermore, physical assessments (i.e., mobility and walking test, handgrip) will be included at baseline. T_1_ is planned as a short questionnaire after the first intervention session including change-sensitive scales to capture immediate intervention effects, whereas T_2_ will serve as a follow-up measurement where most parts of the baseline questionnaire (excluding sociodemographic variable) are repeated. T_3_ and T_4_ are short telephone-based interviews with focus on personal resources. An overview of the used scales and assessments at each measuring point is outlined in Table 1.

**Table 1:** Outcome Measure of the MOBILE trial

Outcome Measure		Operationalisation (Type of assessment)	Times of assessment					Ref
**Main Outcome**			T_0_	T_1_	T_2_	T_3_	T_4_	
Time out-of-home	GPS tracker for seven consecutive days		S	S	S	-	-	[25]
**Secondary Outcomes**								
Life-Space parameter	Aggregated GPS data (i.e. convex hull)		S	S	S	-	-	[25]
Self-perceived life-space	LSA-D		H	H	H	T	T	[6]
Physical activity	IPAQ		H	H	H	-	-	[29]
Depressive symptoms	GDS-12R		H	-	H	-	-	[30]
Health Status	SF-12		H	H	H	-	T	[32]
Frailty	Frailty-phenotype Fried		H	-	H	-	T	[34]
**Mediator variables**								
Goal attainment	Selected items from SOC-questionnaires		H	H	H	-	-	[46]
Habits	4 items from SRHI		H	H	H	-	-	[47]
Lifestyle chance	Action and coping planning, adapted from Schwarzer et al. 2008		H	H	H	-	-	[48]
Balance confidence	ABC-6		H	-	H	-	-	[49]
Health literacy	HLS-EU-Q16		H	-	H	T	T	[50]
Views on ageing	Adapted from DEAS survey		H	-	H	-	-	[51]
Connection to place	Selected items from housing enabler questionnaires		H	-	-	-	-	[52]
Access to health care	Own items		H	-	-	-	-	-
Utilization of health care	Own items		H	-	-	-	-	-
Connection to neighbourhood	Selected items from DEAS survey		H	-	-	-	-	[53]
Social network	Adapted from SHARE survey		H	-	H	-	-	
Loneliness	UCLA Three-Item Loneliness Scale		H	-	H	-	-	[54]
Social Participation	Own items, adapted from SHARE survey		H	-	H	T	T	[51]
**Covariates**								
Sociodemographic data	Age, gender education level, level of care, living arrangements		H	-	-	-	-	-
Physical performance	DEMMI		H	-	H	-	-	[55]
Hand grip	Gauged Hand grip dynamometer, Sahean SH5001		H	-	H	-	-	-
History of falls	Single-item question		H	H	H	T	T	-
Utilization of assistive devices	Own questions, adapted from Baker, 2003		H	-	-	-	-	[6]
Cognition	Mini-Cog		H	-	-	-	-	[56]
Corona	Covid-19 history, vaccination status, anxiety, impact on daily life (own items, single-questions)		H	-	-	-	-	-
Living surroundings, observation by interviewer	Type of house, stairs in front or within the building, pavement		H	-	-	-	-	-

*H,* Home based interview CAPI, *S* self-assessment of GPS-Data with study mobile phone, *T* Telephone-based interview *CATI*, *T*_0_ Baseline measurement, *T*_*1*_ one months after baseline, *T*_*2*_ three months after baseline, *T*_*3*_ 6 months after baseline, *T*_*4*_ 12 months after baseline, *Ref*. reference, *GPS* Global Positioning System, *LSA-D* German version of the University of Alabama Life-Space Assessment, *IPAQ* International Physical Activity Questionnaire, *GDS-12R* short residential Version of the Geriatric Depression Scale, *SF-12* The Short Form Health Survey, *SOC* Selection, Optimization, and Compensation, *SRHI* Self-Report Index of Habit Strength, *ABC-6* Activity-specific Balance Confidence scale *HLS-EU-Q16* Health Literacy Scale, 16 items, *DEAS* German Ageing Study / Deutscher Alterssurvey, *SHARE* The Survey of Health, Ageing and Retirement in Europe, *DEMMI* De Morton Mobility Index, *Mini-Cog* Screening for Cognitive Impairment in Older Adults

**Fig. 1:**
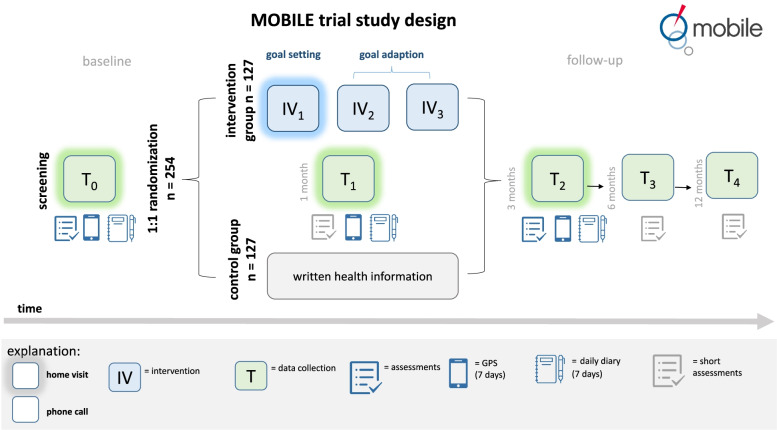
Design of the MOBILE trial

All participants will be equipped with needed technology to track the GPS coordinates and activity diaries measuring out-of-home mobility at three points in time (as outlined in Fig. 1 ) for seven consecutive days each. GPS data is measured using a study mobile phone (ZTE Blade A5) with the “GPS.Rec2.0”-App as the only working application. The app was developed solely for the purpose of this trial and was tested and adapted as part of piloting work. “GPS.Rec2.0” is a mere data collection app and there is no interaction with the study participant. GPS location is recorded every ten seconds with an accuracy of up to 25 m. A detailed description and availability on the technical development of the “GPS.Rec2.0” application is going to be published elsewhere.

The participants in the intervention condition will receive motivational counselling (i.e., one home visit and up to two phone calls) from a trained physiotherapist with the focus of individual mobility goals. Participants in the control condition will be receiving written information on health and mobility.

#### Adaptions due to Corona-pandemic

As the Covid-19 pandemic is both unpredictable and dynamic, changes in the execution of the intervention and questionnaires at T_0_, T_1_, and T_2_ may become necessary. If the situation requires it, we will switch to remote option for the aforementioned tasks. To minimize the risk of infections in general, all contacts with participants will take place in compliance with hygiene regulations of Charité – Universitätsmedizin Berlin and federal state of Brandenburg. Key components of the MOBILE hygiene concept include full vaccination of study personal, wearing FFP2-masks, sanitizing, and ventilation at scheduled intervals. Although protocol deviations may occur due to the pandemic, the main components of the trial, i.e., assessment of study variables and delivery of the intervention will still be feasible, albeit with adjustments, such as telephone instead of personal interviews.

### Participants, interventions, and outcomes

#### Study setting

The MOBILE trial will be conducted in the Havelland region, a rural area in Brandenburg, in the eastern part of Germany next to Berlin, Germany. The region Havelland is characterized by mainly flat landscape, vast areas of forest land, stretches of water, and farming, and a generally low population-density with about 150,000 inhabitants. The study centre is located at the Institute of Medical Sociology and Rehabilitation Science, Charité – Universitätsmedizin Berlin.

#### Eligibility criteria

Participants will be included, if they fulfil all inclusion criteria and none of the exclusion criteria, which are as follows. Inclusion criteria are:

- living in region Havelland, Brandenburg, Germany,
- 75 years or older
- community-dwelling
- having sufficient mobility (i.e., being able to move autonomously or with walking devices and/or little help of others),
- ability tfo give informed consent
- are willing to participate.
  Exclusion criteria are:
- severe cognitive impairments, that prevent living independently
- acute severe events within the past 4 weeks (e.g., falls, operation), that influence out-of-home mobility
- insufficient understanding of German language,
- severe impairment in vision or hearing that will avert participant’s ability to follow the instructions of the study personnel
- reasons of exclusion will be recorded and reported.

#### Intervention

**Fig. 2:**
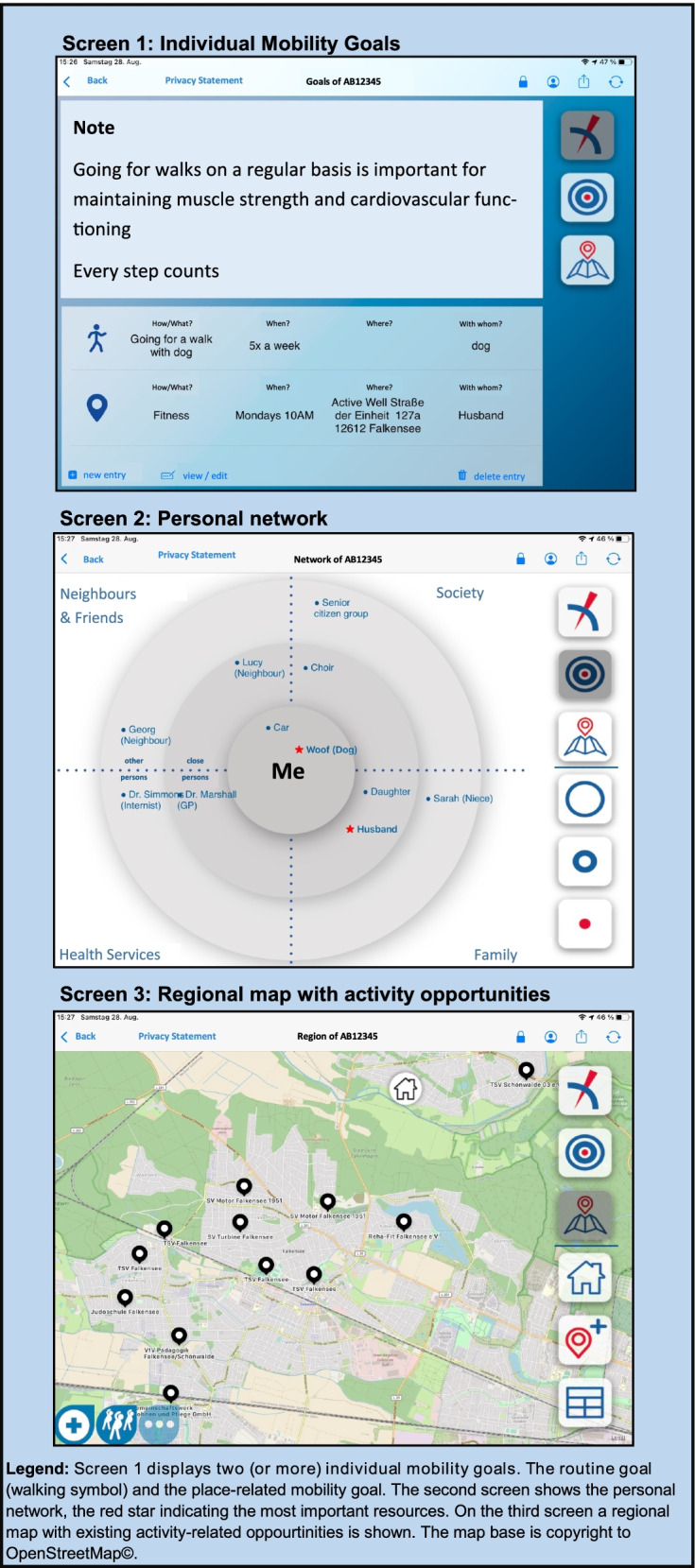
Outline of the Mobile-Intervention-App

The intervention is designed as a tablet-assisted motivational counselling intervention to enhance out-of-home mobility. Summarized it consists of one goal setting and two goal adaption sessions delivered by either one of the two physiotherapists of the study group. All sessions are complemented by the “MOBILE-Intervention-App” (iOS) which is operated by the physiotherapist and works as a documentation and visualization tool. The app was developed by our study team to assist needs of the intervention performance and screenshots of the three main screens of the MOBILE-Intervention-App are depicted in Fig. 2 . Main goal of this intervention is to set and adapt individual goals (e.g., going for a walk, participating in group activity) to foster out-of-home mobility of the participant. All sessions are delivered by the same physiotherapist to rule out information deficits. Couples can take part in the study and are allocated in the same group with each partner receiving separate assessments (T_0_-T_5_). Regarding the intervention, however, there is no strict protocol and couples can either participate together or separately according to their personal preferences and needs. Even when a couple is choosing a joint intervention, mobility goals can still be personalised for each partner and individual profiles are generated in either way. Details of the intervention sessions are described in the following sections and are further classified according to the Behavioural Change Technique Taxonomy (BCT v1) [21]. The BCT Taxonomy was developed by Michie et al. and can be used as a guidance for comparable reporting of complex behavioural interventions. We expect no harms of the intervention and there will be debriefing after the trial.

#### Goal setting (IV_1_)

After the introduction, overview of the study scope, and relationship establishment, the importance of mobility in old age in terms of social, physical, and mental benefits will be highlighted by the physiotherapist *(BCT: information about health consequences 5.1, information about social and environmental consequences 5.3; see* [21])*.* Both physiotherapists are trained on activating positive und relativizing negative views on ageing and mobility by reframing arising negative automatic thoughts and by representing a general age-appreciating attitude *(framing/reframing 13.2)* [22, 23]. The training consisted of extensive literature research, peer-to-peer counselling, and development of a “learning guide”. The latter is composed of a handbook that will help guiding through the sessions and will be amplified and adjusted by the study group during the scope of intervention.

The main objective of the first session is the setting of individual mobility goals *(goal setting (behaviour) 1.1, goal setting (outcome) 1.3)* in relation with social network analysis *(social support (practical) 3.2, social support (emotional) 3.3)* and linking regional services and opportunities for mobility *(social support (unspecified) 3.1, action planning 1.4).* This will be done by firstly filling out the personal network map (Screen 2) to illustrate existing resources. In a second step, a regional network map with existing opportunities for activity engagement is demonstrated (Screen 3) to point out environmental/social resources and link them with the planned goals (Screen 1). The database underlying the regional network map was generated by the study group prior enrolment and is updated on a regular basis. After illustrating both personal and environmental/social resources, individual mobility goals are discussed and recorded in the intervention app (Screen 1). It is planned to intended at least two distinct mobility goals: One of them being a routine goal (e.g., going for a walk every Monday morning) *(action planning 1.4, habit formation 8.3*) and the other one being a goal with a specific destination (e.g., going to a museum, *action planning 1.4*) that is asked to be routine as well, yet this is not mandatory. It is important to note that a defined goal can also be maintaining the current mobility status (*focus on past success 15.3)*, as maintaining mobility is important for prevention of frailty despite possible decline in physical capacities in old age. Any foreseeable barrier that may impede goal achievement will also be discussed (e.g., loss of physical capacity, pain, environmental challenges) *(action planning 1.4)*. Goal selection and strategies to attain the goals will be set and explored based on the assumption of the selection *(goal setting (behaviour) 1.1, goal setting (outcome) 1.3, action planning 1.4)*, optimization *(commitment 1.9)*, and compensation *(coping planning 1.2)* (SOC) approach [24]. All screens can be printed out directly during the session by using a battery-printer with internal Wi-Fi to ensure that printing works everywhere (i.e., Canon Pixma TR150). This will enhance recognition of the first session, as the participants can keep record of the conversation. The first session will take approximately 60 to 90 min and a new appointment for the following session will be scheduled individually within four weeks.

#### Goal adaption (IV_2_ and IV_3_)

The booster sessions will be performed as telephone interviews and focus on goal adaption and habit formation. The foundation of these sessions are the defined mobility goals that have been established in the first interview. For easy and simple guidance, the outlines of the personal network map and mobility goals that were printed out at the first intervention session are used. The personal network map and each goal will be individually reviewed and discussed regarding whether it was well chosen or if any unexpected barriers occurred *(review behaviour goal(s) 1.5, feedback on behaviour 2.2)*. Goal adaption will then be individually and flexibly adjusted to personal circumstances *(action planning 1.4, coping planning 1.2)*. Moreover, these sessions should not only focus on barriers or optimization but also affirm and encourage *(focus on past success 15.3)* on goals that worked well, especially when the goal is maintaining the current mobility status. A further focus of the follow-up sessions is to transfer the chosen goals into habitual routines of out-of-home mobility (8.3 habit formation). A documentation of the goal adaption session is sent to the person´s home if major modifications have been made. Time needed for each goal adaption session will be about 30 to 45 min.

#### Control condition

The control condition will receive a booklet containing general health information *(information about antecedents 4.2, information about health consequences 5.1)* and advice on how to get more active *(social support (unspecified) 4.1)*. This booklet contains eight pages in A5 format and besides general advice on healthy ageing also includes a page featuring local stakeholder that offer mobility-related (group) activities and one page for phrasing individual goals *(goal setting (behaviour) 1.1)*.

Critical events will be reported for both intervention and control condition. In order to ensure participation adherence the study team contacts participants via phone on a regular basis.

#### Outcomes

## Primary outcome

The primary outcome variable is “time out-of-home” (TOH) based on the objective GPS measurement and meaning the total time spent outside of one´s home radius (i.e. 50 m) per day. TOH can be measured in minutes or percentage. A day in this connection is defined from 3:00 AM to 02:59 AM to include trips than last longer than midnight and as recommended in literature [25, 26]. GPS data is monitored on seven consecutive days at T_0_, T_1_, and T_2_ respectively. An overview of all outcomes, assessments and time of measurement is demonstrated in Table 1.

## Secondary outcomes

Aggregated GPS data: Raw GPS data can be aggregated to certain parameters to describe someone´s life-space. Here we use “convex hull”, a descriptive measure to specify the area of all daily GPS fixes [25]. We will further acquire other variables (such as time first moved, standard deviational ellipse, maximum distance of home) that have been identified as important in literature [7, 25, 27]

Self-perceived life-space mobility: This is measured using the German version of the University of Alabama Life-Space Assessment [8], that has been translated and validated for the use in urban and rural settings by our study group prior to the MOBILE trial [28]. The life-space is assessed both at times of assessment (T_0_, T_1_, and T_2_) on a weekly basis and during the seven-day GPS tracking on a daily basis as part of the activity diary.

Physical activity: Physical activity is assessed using the International Physical Activity Questionnaire (IPAQ) in the short version for older adults [29]. This short version consists of four questions addressing both the number of times per week and minutes per days of strenuous activities, moderate activities, walking, and sitting. A score is then generated and the categories of high, moderate, and low level of activity are composed.

Depressive symptoms: Depressive symptoms are addressed using a validated shorter version [30] of the Geriatric Depression Scale [31]. In the shorter version for residential study population, 12 dichotomous questions are asked and a score is then evaluated.

Health status: The international approved Short-Form 12 (SF-12) [32] of the 36-item short-form health survey (SF-36) [33] is used to measure the subjective overall health status for the last seven days.

Operationalisation of Frailty: The used Frailty phenotype approach is based on a cluster of five clinical criteria (reduced muscle strength, slow gait speed, exhaustion, weight loss, low level of physical activity) developed by Fried et al. in 2001 [34]. This phenotype is one of the most popular tools to evaluate frailty, predict adverse health outcomes and distinguishes well between three different stages of frailty (robust, pre-frail, frail) in older adult [34, 35]. To rate the physical criteria of the phenotype, functional tests are performed using the SAHEAN 5001 hand grip dynamometer to assess handgrip strength [34] and a 3 m walking test in preferred speed to measure gait speed [36]. To assess the criterion of exhaustion two questions of the Centre for Epidemiological Studies depression (CES-D) scale are used like in the original publication by Fried et al. and weight loss was defined as unintentional weight loss of ≥ 5 KG in the prior year [34]. The criterion of low physical activity is retrieved from a single item question of the validated SHARE-frailty phenotype Instrument [37].

Mediators and covariates are also outlined in Table 1 and contain personal, social, and environmental resources.

### Sample size

For the sample size calculation, results of previous intervention studies for increasing mobility in older adults were considered [38, 39]. A relevant change in mobility is considered as an increase in mobility of 15% after three months (Life Space *M* = 65.4 (*SD* = 20.1) + 15% = 75.2 [40] in comparison to the control condition (1:1 ratio, power 1 – beta = 95%, alpha (two-sided) = 5%, dropout = 15%). Based on these presumptions an online tool (powerandsamplesize.com) has been used to calculate sample size, which is stated as follows: *N* = 110 + 15% dropout in each condition and therefore a total sample size of *N* = 254 (*N* = 127 in each condition) is needed.

### Recruitment of participants

A number of complementary strategies will be employed for the recruitment of participants. First approaches include a newspaper article, displaying of study information flyer on designated spots (e.g., supermarkets, pharmacies, health or public institution), and recruitment via our stakeholder network. Further steps will include postal invitations to all citizen of Havelland aged 75 and older (data has been provided by the public register for the purpose of this trial), presentation of our trial on various occasions (e.g., sport groups, social activities, public events), internet magazine articles, and advertisement. We implemented a website, a flyer, and posters to complement our recruitment strategy. Recruitment success of each path and strategy will be tracked and readjusted if applicable. Once an interested person contacts the MOBILE study team, the initial screening for eligibility (age, place of living, mobility) will be performed via phone, where questions can be asked and the further course of the study will be explained. Those, who are interested and eligible, will then receive the study information letter, consent forms, and hygienic concept via post. Informed consent form will be collected at the baseline measurement (T_0_) once the initial screening is confirmed by the study nurse. Study participants receive no financial compensation, however all participants get to keep the bumbag with the study emblem printed on, that was given to them with the GPS-tracker.

### Assignment of Interventions

#### Allocation and blinding

After baseline assessment, participants included in the study will be randomly assigned to intervention or control condition. Due to practical implications a couple will always be assigned to the same group. Randomization to either intervention or control condition is performed computer-assisted with 1:1 ratio by using the package “blockrand” (block randomization with block sizes of 4, 6, and 8 as well as strata) using R version 4.0.4. Strata are as follows: older (> 80 years) / younger (75 – 80 years) and living in the centre of one of the three biggest towns in Havelland (i.e. > 15.000 inhabitants) / not living in the centre of one of these three biggest towns). The centre of every single town was defined individually by criteria of buildings in closed development, provided services, and infrastructure [41].

Allocation is concealed until after baseline measurement, when the study nurse uncovers the allocation. Randomization sequence, enrolment of participants, and allocation of the participants are strictly separated. The study nurse and participants are blinded during the baseline measurement. Since our proposed trial includes a home visit (in comparison to a written health information) blinding for study personal or participants cannot fully be maintained over the further course of the study. GPS data will be processed and analysed by data scientists at TU Berlin who will be blinded throughout the whole study period.

### Data collection, management and statistical analysis

#### Data Collection

All data is collecting using a Charité-owned laptop and data is recorded in a Microsoft Access 2016 database to ensure easy exporting to SPSS v25 (IBM, Cary, Ind.) where statistical analysis will be primarily performed. GPS data of the participants is stored on the study mobile phone and is then uploaded by the study team on Charité premises to the TU Berlin server to ensure secure data handling.

#### Data management

The collected data is regularly checked upon consistency, accuracy, and completeness. Since the data is solely collected digitally (except activity diary) an automated back-up system (once a week) has been implemented. GPS data is also regularly reviewed for accordance with the activity diary. Only the study team will have access to the data, as defined in the data protection concept that was approved by ethics committee and clinical trial office.

#### Statistical analysis

Raw GPS data will be checked and aggregated by a computer scientist of the TU Berlin und will be prepared for further analyses. All analyses will be performed using SPSS v25 (IBM, Cary, Inc.) and in R Project using the intention-to-treat principle. For the demographic variables, means, standard deviations and frequencies will be calculated, and distribution will be checked. Medians and interquartile ranges will be reported for non-normally distributed data. To test the primary hypothesis, multilevel modelling will be used to estimate the group difference in the primary outcome across T_0_-T_2_ as predefined in the power analysis. Covariance analysis will also examine possible mediating variables declaring any relationship between out-of-home mobility and individual resources. The level of significance will be set to p < 0.05 (two-sided) and 95% confidence interval will be reported. Multiple imputations using a package (e.g., AMELIA II) will be applied to handle missing data if necessary.

#### Trial status

Rolling recruitment started on 7 June 2021 (i.e., first participant in) for a duration of presumably 12 months, meaning that the last participants included in the study will have their baseline assessment in June 2022 und their last assessment one year later in June 2023 marking the end of the field phase. Recruitment can be extended by up to six months, if the required number of participants has not yet been reached. The funding period for this trial is until February 2024.

## Ethics and dissemination

### Research ethics approval

This trial was approved by the Ethics Committee of Charité – Universitätsmedizin Berlin (14/05/2020, EA1/052/20).

### Protocol amendments

Relevant changes of protocol will be stated in the trial paper.

### Consent or assent

The participant of the MOBILE trial will receive comprehensive information on the research project and trial and written informed consent will be obtained by study nurse prior to data collection.

### Declaration of interests

The authors declare that they have no competing interests.

### Access to data

Data collected during the study will be available under the search term “MOBILE” at the institutional refubium repositorium after study is completed and in an aggregated and anonymized form, whereas anonymity cannot be guaranteed at a raw data level.

### Dissemination policy

Publication of trial results are planned to be published in peer-reviewed journals. As for public audience results will be disseminated through low-threshold channels such as local talks, newspaper articles or interviews. Both codes of the developed apps (“GPS.Rec.2.0” and “MOBILE-Intervention-App”) are planned to be available as open source.

## Discussion

The proposed MOBILE trial will advance the knowledge on the effects of a motivational counselling for fostering out-of-home mobility in community-dwelling older adults in rural areas. Furthermore, we will achieve a deeper understanding of underlying individual, social and environmental resources for out-of-home mobility and their interconnection in this population. Approaches that at least focusing on the individual resources have been tested in Finland (i.e. COSMOS Trial, AGNES Study) [11, 16, 42], and are currently under investigation [42]. However, these trials specifically looked into physical activity, falls, and healthy aging in general and therefore have merely incorporated self-reported measurements of out-of-home mobility. In contrast, the main outcome of the proposed MOBILE Trial is out-of-home mobility based on GPS data, which are to become state-of-the-art of measuring mobility [17, 43]. This includes derived specific mobility indicators (e.g., time spent out-of-home, maximal distance from home, convex hull) [25, 44, 45] and the linkage of GPS and movement data with geographic information systems (GIS). Another notable benefit of objective mobility measure is that we can match participant’s individual goals with their real performance within the interval of assessment. As the out-of-home mobility is largely represented by moving through different life-spaces we use the Life-Space Assessment [8] and validated the instrument for the German population [28]. The ecological approach assures high accessibility and easy implementation, thus maximizing the benefits for the people within the region.

## Data Availability

Data collected during the study will be available upon reasonable request

## Abbreviations

ABC: Activities-specific Balance Confidence scale
BCT: Behavioral Change Technique
BMBF: Bundesministerium für Bildung und Forschung (Federal Ministry of Research and Education)
CAPI: Computer-assisted personal interview
CATI: Computer-assisted telephone interview
DEAS: Deutscher Alterssurvey (German Ageing Survey)
DEMMI: De Morton Mobility Index
DRKS: Deutsches Register Klinischer Studien (German Registry of Clinical Trials)
GDS: Geriatric Depression Scale
GPS: Global Positioning System
GIS: Geographic Information System
HLS: Health Literacy Scale
IPAQ: International Physical Activity Questionnaire
LSA-D: German Version of Life-Space Assessment
SHARE: The Survey of Health, Ageing and Retirement in Europe
SF-12: The Short Form Health Survey
SOC: Selection, Optimization and Compensation
SRHI: Self-Reported Habit Index
TOH: Time out-of-home
TU Berlin: Technische Universität Berlin
